# Comparison of Non-Operative Mesotherapy and Surgery in the Management of Superficial Lipomas

**DOI:** 10.21315/mjms2021.28.1.7

**Published:** 2021-02-24

**Authors:** Kamal Kataria, Meghna Venkatesh, Sunil Chumber, Yashwant Rathore, Anurag Srivastava, Anita Dhar, Piyush Ranjan, Rajni Yadav, Surabhi Vyas, Priyanka Naranje

**Affiliations:** 1 Department of Surgical Disciplines, All India Institute of Medical Sciences, New Delhi, India; 2 Department of Pathology, All India Institute of Medical Sciences, New Delhi, India; 3 Department of Radiodiagnosis, All India Institute of Medical Sciences, New Delhi, India

**Keywords:** deoxycholate, lipoma, mesotherapy, phosphatidylcholine

## Abstract

**Background:**

Lipomas are benign adipocytic tumours. Surgical excision is the gold standard for treating such lipomas, but it results in unaesthetic scarring.

**Methods:**

A total of 126 patients were randomised into two groups. The patients in Group A underwent mesotherapy (*n* = 66) and those in Group B underwent surgery (*n* = 60). The patients in Group A group received six sessions of mesotherapy treatment at 2-week intervals. Both groups were followed up for 12 weeks, during which they were assessed for complications arising from treatment, reduction of the size of the lipoma and cosmetic outcomes.

**Results:**

The overall mean age of the patients was 32.93 (± 10.1) years old and the mean volume of the lipomas was 2.29 (± 3.8) mL. A 55.86% (*P* = 0.0032) mean reduction in the volume of lipomas was noted in the patients who received mesotherapy, while one patient showed a gain of 16% by volume. The patients in Group A (cosmetic score ≥ 4: 63%) were happier with the treatment than those in Group B (cosmetic score ≥ 4: 21%).

**Conclusion:**

Our findings indicate that mesotherapy modestly reduces the volume of lipomas with very few and minor complications and excellent cosmetic outcomes.

## Introduction

Lipomas are adipocytic tumours which can arise in any part of the body (1). They are most often benign asymptomatic neoplasms but can cause symptoms as a result of compression or displacement of the surrounding structures. They are generally well encapsulated and homogenous but may develop calcification and haemorrhage due to trauma. Their diagnosis is mainly clinical, but an ultrasonography and fine-needle aspiration cytology (FNAC) may be required to confirm the diagnosis. The common indications for treatment of lipomas are cosmesis, progressive enlargement and fear of malignancy.

The treatment of a lipoma commonly includes simple excision beyond its capsule. This procedure usually results in scarring and may also be associated with surgical complications such as haematoma and seroma formation (2). Newer minimally invasive methods such as liposuction, laser lipolysis and endoscopic removal have better cosmetic outcomes than surgical excision. Most of these minimally invasive methods involve making incisions of varying sizes over or away from the lipoma. Mesotherapy (injection lipolysis) has an advantage over these surgical procedures with respect to cosmesis because it does not require an incision of any sort. The term ‘mesotherapy’ was coined to describe the treatment of pathologies of tissues arising from the mesoderm. This treatment modality soon expanded to include the treatment of cellulite in obese patients when several trials reported the localised loss of adipose tissue by subcutaneous injections of phosphatidylcholine and sodium deoxycholate (3, 4). Sodium deoxycholate, the active component in mesotherapy, induces the destruction of fat cells in a non-specific fashion due to its detergent action (5, 6).

In this randomised controlled trial, our primary objective was to compare surgery and mesotherapy with respect to cosmesis and assess the reductions in the size of the lipomas in the mesotherapy group. Although a few pilot studies conducted similar trials, the data remains insufficient, and to date there have been no studies performed on the Indian population, as this study was.

## Methods

**Figure 1:**
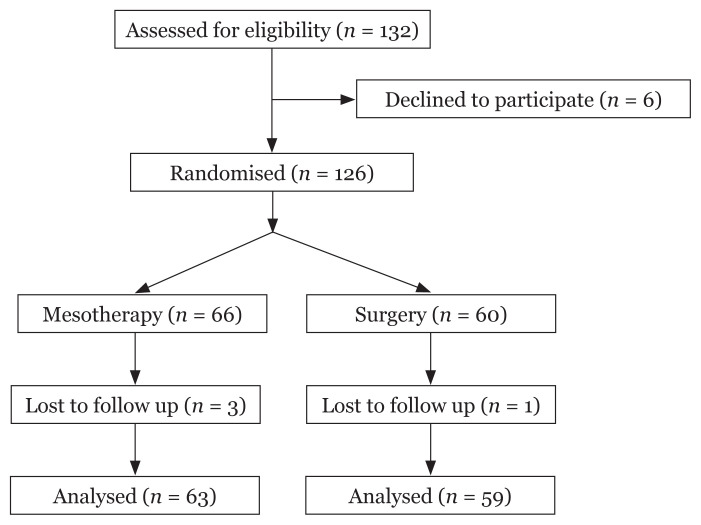
Consort diagram

**Figure 2:**
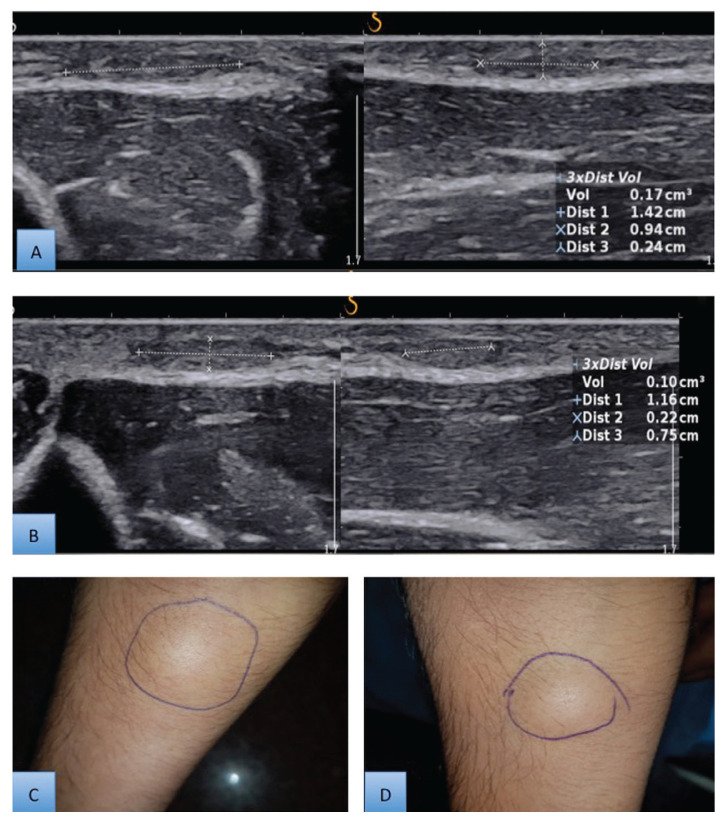
Measurement of response in mesotherapy group on ultrasonography. **A**. Pre-treatment dimensions of lipoma on ultrasonography; **B**. Post-treatment dimensions of lipoma on ultrasonography; **C**. Pre-treatment appearance of lipoma on inspection; **D**. Post-treatment appearance of lipoma on inspection

This randomised controlled trial was conducted in our department from January 2017 to January 2019. Approval to conduct the study was granted by the Institute Ethics Committee. Patients aged 18 years old or older with superficial subcutaneous swellings already confirmed to be lipomas by FNAC and less than 5 cm in size (dimensions determined by ultrasonography) were included in the study. Patients with a known allergy to any ingredient in the injection or with a current infection of the overlying skin were excluded from the study. Pregnant and lactating mothers were also excluded from the study. A total of 132 patients were enrolled in the study, but only 126 were randomised into the mesotherapy and surgery groups after evaluation and consent (Figure 1). Block randomisation was used to divide the patients into two groups. All patients were assessed for pain, swelling, erythema and any additional reactions during the first 24 h. A total of six injection therapy sessions were administered at 2-week intervals to the patients in mesotherapy group (Group A). The patients’ response to treatment was assessed by ultrasonography at the end of 12 weeks in the mesotherapy group (Figure 2).

**Figure 3:**
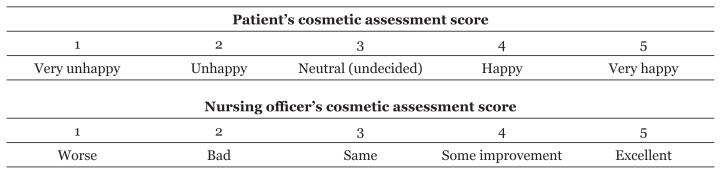
Method of cosmetic assessment

At the end of treatment, patients in both groups were compared in terms of pain during therapy, patient satisfaction, cosmesis and complications. Pain scoring was conducted using the visual analog scale (VAS). Patient satisfaction was measured on a 5-point Likert scale, where 1 denoted ‘strongly disagree’ and 5 denoted ‘strongly agree’. The success of any therapy was defined as a score of ≥ 4. Similarly, cosmetic assessment was conducted using a 5-point Likert scale administered by a nursing officer, both pre- and post-treatment (Figure 3). A score of ≥ 3 indicated that the treatment was successful. All patients in both groups were assessed for complications such as pain, swelling, rash and redness. The patients in Group B, who underwent surgery, were also observed for surgical site infections, haematoma or seroma.

### Mesotherapy

**Figure 4:**
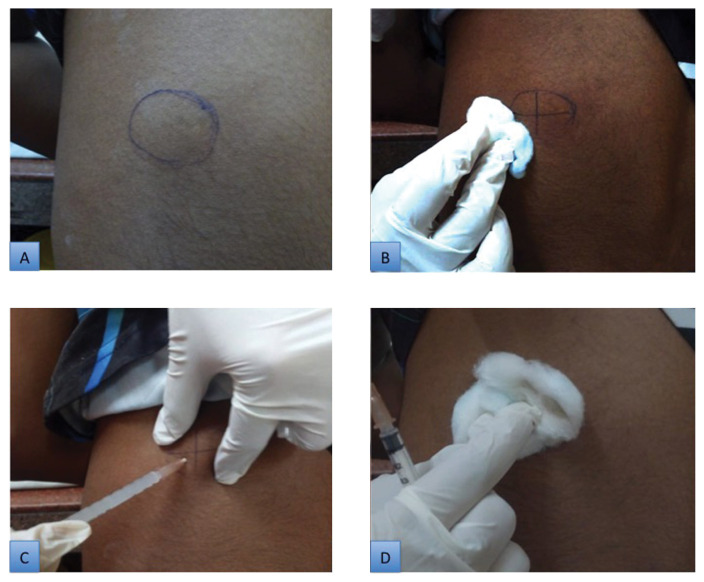
**A**. Marking of the lipoma; **B**. Cleaning of site with spirit; **C**. Intralesional injection; **D**. Pressure to local site

A 26-gauge, half-inch needle attached to a 2 mL syringe was used to inject the solution (a mixture of phosphatidylcholine and sodium deoxycholate) transcutaneously into the lipoma without any topical or local anaesthetic. In India, this solution is available as ‘+Lipolab’. Patients were treated with a volume of solution in millilitres equal to half of the largest dimension of the lipoma in centimetres. In cases where a lipoma measured more than 1 cm, the total area of the lesion was divided into small grids (~1.0 cm apart). A total of 0.4 cc of solution was injected into each grid using the pinch/ pull technique (Figure 4). Care was taken not to inject any solution while the needle was being withdrawn, to ensure that none was deposited intradermally. Following each injection, pressure was applied directly to the site of injection for several seconds to prevent local bleeding.

### Statistical Analysis

All the statistical analyses were carried out using the statistical software STATA version 14.0. Data were expressed as frequencies and percent values. Descriptive statistics such as mean, median, standard deviation (SD), range and interquartile range were calculated. Comparisons of mean or median values of the dimensions and volumes of the lipomas pre- and post-treatment were conducted using the paired *t*-test, as appropriate.

## Results

The study included 126 patients who met the enrolment criteria. Four patients dropped out from study during follow-up. The remaining 63 patients in the mesotherapy group and the 59 in the surgery group were analysed. Their overall mean age was 32.93 (± 10.1) years old (range 18–62 years old). Ninety-six patients were aged between 20 and 40 years old. There were 24 (20%) female and 98 (80%) male patients. The two groups were comparable in terms of patients’ ages and gender and the size of lipomas (Table 1). The most common location of the lipomas overall was an upper limb (78.69%), followed by the trunk (15.58%). A total of 39 patients were found have multiple (3 or more) lipomas: 19 of these patients were in the mesotherapy group and 20 were in the surgery group.

The length, breadth, height and volume of each lipoma, measured using sonography before any intervention (pre-treatment dimensions), were comparable in both groups (Table 1). Of the 63 patients, 55 completed six sessions of treatment. There were significant reductions in the length, breadth and height of their lipomas after mesotherapy (*P* < 0.001). Significant reductions in volume were also achieved, with an overall *P*-value of 0.0032 (Table 2). Other features such as shape and vascularity showed no changes, but the lipomas did become firm to the touch after mesotherapy.

Patients who received mesotherapy were statistically more satisfied and happier with regard to cosmesis compared to those patients who underwent surgery (*P* > 0.007) (Table 3). When the two groups were compared in terms of pain and swelling, it was observed that the patients in the mesotherapy group experienced significantly less pain (VAS score ≤ 3) than those who underwent surgery (VAS score ≥ 6). There was no need to administer analgesics to the patients in the mesotherapy group. Similarly, minimal swelling was observed at the injection sites for all patients; any swelling present resolved completely and spontaneously within 48 h. In the surgery group, however, five patients developed significant swelling at the site of the incision and were evaluated for a haematoma/ seroma.

**Figure 5:**
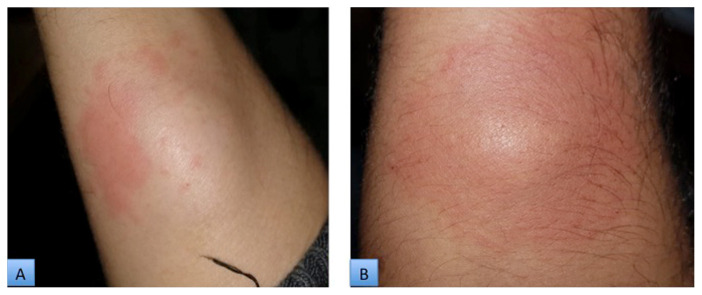
Appearance of rash after mesotherapy

The only worrisome complication observed in the mesotherapy group was rash and redness (Figure 5), which responded to the oral antihistamine cetirizine (5 mg) and the application of a topical emollient (coconut oil or liquid paraffin); all cases resolved within 3 days. Five patients developed this complaint, and it caused two of them to drop out of the trial. Although none of the patients in the surgery group experienced complications such as rashes, five patients did develop surgical site infections and five developed a haematoma or seroma at the site of the incision. The surgical site infections were resolved after a 1-week course of oral antibiotics, but the patients who developed a haematoma or seroma had to undergo aspiration in addition to 1 course of oral antibiotics. All 10 of these cases resolved within a week and there was no recurrence.

## Discussion

Lipomas are the most common soft-tissue tumours encountered by surgeons. The incidence of lipomas is underreported, probably due to the asymptomatic nature of these tumours, but they are estimated to occur in 1% of the population, with an estimated prevalence rate of 2.1 per 1000 tumours. The incidence of lipomas peaks in the fifth and sixth decades of life, and they are slightly more common in men (7). The aetiology of lipomas is unknown. Several theories have identified obesity, trauma and genetics as possible contributing factors to their development. Cytogenetic studies have revealed an association between translocations and inversions of regions 12q13-15 and 6p-13q with the formation of lipomas (8).

Lipomas are benign lesions with no risk of malignant transformation, so they can be managed conservatively. Surgical excision, which at present remains the gold standard treatment, is offered when a lipoma is found to be cosmetically unappealing (9). However, surgical excision is associated with unaesthetic scarring, pain, risk of surgical site infection and, rarely, recurrence. Due to these negative effects, alternative methods of treating lipomas have recently been developed. Excision via mini incision/squeeze technique, laser lipolysis, liposuction, endoscopic resection, and injection lipolysis (mesotherapy) are the other treatment modalities available, especially for patients with multiple lipomatosis. While most of these alternative treatments also involve making incisions of varying sizes over or away from the lipoma, mesotherapy does not, thus giving it an advantage over other procedures with respect to cosmesis.

Michel Pistor is credited with developing mesotherapy in France in 1952, but the roots of mesotherapy can be traced back to 2000 BC in China (10). Pistor coined the term mesotherapy to describe the treatment for pathologies of tissues originating from the mesoderm. Mesotherapy initially referred to intradermal injections, but today it encompasses injections into the epidermis and dermis, as well as subcutaneous and regional injections. Mesotherapy has been advocated for treating chronic pain, arthritis, psoriasis, vascular diseases, etc. Mesotherapy with phosphatidylcholine and deoxycholate has been recently introduced for aesthetic purposes to treat cellulite (11). The downside of mesotherapy is that there are as yet no standardised protocols or formulae for the drugs administered.

In the literature, only five studies have reviewed the use of mesotherapy to manage lipomas as described in Table 4 (12–16). The negative points of all these studies were the very small sample populations used and the lack of a standardised protocol for the treatment. Neither the number of sessions nor concentration of the mesotherapy solution was kept constant. Bechara et al. (12) were the only researchers to evaluate patient satisfaction, using a VAS for this purpose. A score of 1 or 2 indicated patient satisfaction: nine patients (90%) were satisfied, while one patient (10%) was not at all happy (12). In our study we assessed patient satisfaction on a numerical scale of 1–5 and an objective nursing officer assessment score. Most patients (63%) were happy with the treatment. No patient expressed the desire to undergo excision following mesotherapy. This is comparable to the patient satisfaction expressed in the studies cited above.

The main complications seen following mesotherapy were mild pain (VAS score ≤ 3), swelling at injection site during the initial 24 h–48 h and rashes over the injection site. Other studies in the literature reported similar complications, except for Hayward et al. (16), who reported hypopigmentation due to steroid injection at 4 months post-treatment. Hayward et al. (16) also reported that three patients (37.5%) developed a recurrence within 2 years; two of these patients received a second injection while the third underwent surgical excision of the lipoma. Rotunda et al. (13) observed that pain and burning sensation were more common with higher concentrations (5%) of deoxycholate.

## Conclusion

Surgery remains the gold standard treatment for lipomas but mesotherapy is a good alternative, especially in cases of lipomatosis. Mesotherapy can be provided on an outpatient basis, does not require anaesthesia, causes significantly less pain and has excellent results with regard to cosmesis.

## Implications and Recommendations

- A study with a longer follow-up period is needed to monitor patients for any increases in size or recurrence of lipoma after mesotherapy
- The drug dosage and number of sessions of mesotherapy need to be standardised

## Figures and Tables

**Table 1:** Pre-treatment dimensions (measured with ultrasonography) of lipomas in both groups

Variable	Mesotherapy *n* = 63	Surgery *n* = 59
Length in cm	2.38 (0.86) (1.1–4.8)	2.26 (0.89) (0.86–4.8)
Breadth in cm	1.28 (0.88) (0.27–3.5)	1.41 (0.83) (0.24–3.25)
Height in cm	1.28 (0.69) (0.46–3.64)	1.1 (0.55) (0.36–2.5)
Volume in mL	2.29 (3.8) (0.23–23.63 )	2.17 (3.51) (0.26–23.63)

**Table 2:** Post-mesotherapy dimensions in mesotherapy group

Variable	Pre-treatment	Post-treatment	*P*-value	Mean percentage reduction
Length in cm	2.38 (0.86) (1.1–4.8)	1.61 (0.78) (0.26–3.2)	0.0000	32.35 %
Breadth in cm	1.28 (0.88) (0.27–3.5)	0.88 (0.78) (0.2–3.2)	0.0000	31.25 %
Height in cm	1.28 (0.69) (0.46–3.64)	0.87 (0.64) (0.2–2.33)	0.0000	32.2 %
Volume in mL	2.29 (3.8) (0.26–23.63)	0.78 (1) (0.10–4.5)	0.0032	55.86 %

**Table 3:** Cosmetic assessment by patient and nursing officer

Patient’s assessment score		

Variable	Mesotherapy	Surgery
Score 1,2	9%	25%
Score 3	28%	54%
Score 4,5	63%	21%

**Nursing officer’s assessment score**		

Score 1,2	9.26%	16.95%
Score 3	18.52%	54.24%
Score 4,5	72.22%	28.81%

**Table 4:** Brief of studies, using mesotherapy for management of subcutaneous lipomas

Treatment	Study	Site	*N*	Outcome measured	% Change in size
Phosphatidylcholine/Deoxycholate	Bechara et al. (12)	Dermal lipoma	30	Cross sectional surface	45.8 % decrease
Deoxycholate	Rotunda et al. (13)	Dermal lipoma	12	Surface area	75% decrease
Phosphatidylcholine/Deoxycholate	Kopera et al. (14)	Dermal lipoma	19	Volume	44% decrease in 13/19 41% Increase in 6/19
Steroid + beta 2 agonist	Redman et al. (15)	Dermal lipoma	10	Volume	50% decrease
Triamcinolone acetonide	Hayward et al. (16)	Superficial and deep lipomas	8	Palpable dimension	60% reduction
